# Gene expression, molecular docking, and molecular dynamics studies to identify potential antifungal compounds targeting virulence proteins/genes VelB and THR as possible drug targets against *Curvularia lunata*

**DOI:** 10.3389/fmolb.2022.1055945

**Published:** 2022-12-12

**Authors:** Himanshu Kamboj, Lovely Gupta, Pawan Kumar, Pooja Sen, Abhishek Sengupta, Pooja Vijayaraghavan

**Affiliations:** ^1^ Anti-mycotic Drug Susceptibility Laboratory, Amity Institute of Biotechnology, Amity University, Noida, India; ^2^ School of Computational and Integrative Sciences, Jawaharlal Nehru University, New Delhi, India; ^3^ Systems Biology and Data Analytics Research Laboratory, Amity Institute of Biotechnology, Amity University Uttar Pradesh, Noida, India

**Keywords:** *Curvularia lunata*, molecular docking, molecular dynamics, bioactive molecules, virulence proteins

## Abstract

*Curvuluria lunata* is a melanized fungus pathogenic to both plants and animals including humans, causing from mild, febrile to life-threatening illness if not well treated. In humans, it is an etiological agent of keratomycosis, sinusitis, and onychomycosis in immunocompromised and immunocompetent patients. The development of multiple-drug-resistant strains poses a critical treatment issue as well as public health problem. Natural products are attractive prototypes for drug discovery due to their broad-spectrum efficacy and lower side effects. The present study explores possible targets of natural antifungal compounds (α-pinene, eugenol, berberine, and curcumin) against *C. lunata via* gene expression analysis, molecular docking interaction, and molecular dynamics (MD) studies. Curcumin, berberine, eugenol, and α-pinene exhibited *in vitro* antifungal activity at 78 μg/ml, 156 μg/ml, 156 μg/ml, and 1250 μg/ml, respectively. In addition, treatment by these compounds led to the complete inhibition of conidial germination and hindered the adherence when observed on onion epidermis. Several pathogenic factors of fungi are crucial for their survival inside the host including those involved in melanin biosynthesis, hyphal growth, sporulation, and mitogen-activated protein kinase (MAPK) signalling. Relative gene expression of *velB*, *brn1*, *clm1*, and *pks18* responsible for conidiation, melanin, and cell wall integrity was down-regulated significantly. Results of molecular docking possessed good binding affinity of compounds and have confirmed their potential targets as THR and VelB proteins. The docked structures, having good binding affinity among all, were further refined, and rescored from their docked poses through 100-ns long MD simulations. The MDS study revealed that curcumin formed a stable and energetically stabilized complex with the target protein. Therefore, the study concludes that the antifungal compounds possess significant efficacy to inhibit *C. lunata* growth targeting virulence proteins/genes involved in spore formation and melanin biosynthesis.

## Introduction

The *Curvularia* genus is one of the major groups of opportunistic human pathogenic dematiaceous filamentous fungi (Alex et al., 2013). Within the genus, *Curvularia lunata* (teleomorph sexual state—*Cochliobolus lunatus*) is in the growing list of emerging fungal pathogens in humans (Giri et al., 2011; Chowdhary et al., 2014), whereas it is reported pathogenic to animals and plants (Beckett et al., 2017; Gupta et al., 2017; Bisht et al., 2018; Liu et al., 2019). The infections caused by *Curvularia* spp. include phaeohyphomycosis, non-dermatophytic onychomycosis, mycetoma, and infections in eyes, nails, sinuses, and wounds (Vineetha et al., 2016). Infections generally occur through direct inoculation of conidia or by inhalation, leaving it susceptible for invasion (Shrivastava et al., 2017). Patients with peritoneal, venous catheters, intravenous drug abusers, and cataract surgery patients are more prone to *C. lunata* infections (Alex et al., 2013).

Azoles (itraconazole and voriconazole) and polyenes (amphotericin B) are antifungals that have widely been used to control invasive human fungal infections for more than 4 decades (Jørgensen and Heick, 2021; Uma Maheshwari Nallal et al., 2021). The clinical use of azoles is of high priority since there are only a few available alternatives for prophylactic and therapeutic treatment of *C. lunata* infections (Chowdhary et al., 2014; Chang et al., 2019). *C. lunata* infections like foliar disease and leaf blight have been reported in plants (Liu et al., 2014; Garcia-Aroca et al., 2018), and to control these infections, a variety of fungicides were used in the crop fields. Excessive use of synthetic fungicides poses selective pressure on cross-kingdom pathogens and impacts antifungal drug resistance (Hof, 2001). Transferability of such drug-resistant isolates from farms to humans through the human–plant interaction stances a critical public health concern (Bengyella et al., 2017) as some of the azole fungicides possess chemical structures similar to medical azole (Snelders et al., 2012).

Natural compounds have received a renewed interest in their use as antimicrobials because of uncontrolled usage of synthetic drugs or fungicides (Nagoor Meeran et al., 2017). These can be exploited in controlling the growth of fungi consequently inhibiting secondary metabolite production. The effect of plant extracts has been investigated on *C. lunata* depicting alteration in growth, sporulation, and secondary metabolite pathways (Ghany TM et al., 2015). Many investigations resulted in the screening of a wide variety of plant species/bioactive compounds for their antimicrobial activities and have revealed structurally unique biologically active compounds (Matasyoh et al., 2007). Their target identification can be approached via direct biochemical assays, molecular studies, or using computational methods. Furthermore, the mechanism of action of compounds can be generated by studying gene expression data in the presence or absence of it.

The pathogenicity of *C. lunata* involves a plethora of virulence factors including melanin pigment (Xu et al., 2007), siderophores (Wang et al., 2013), hydrophobins, and non-host-specific toxins (Gao et al., 2012). A number of genes and proteins are involved in these pathways including *brn1*, *clpks18*, *clvelB*, and *clm1* which are involved in melanin biosynthesis, hyphal growth, sporulation, non-ribosomal peptide synthetase, and cell wall integrity (Rižner and Wheeler, 2003; Gao et al., 2012; Fu et al., 2022). Melanin deposition in the cell wall of fungus protects it from host macrophage attack and phagolysosome oxidative burst of neutrophiles (Rižner and Wheeler, 2003; Tóth et al., 2020)*.* Multiple genes are involved in the melanin production pathway of the fungus directly or indirectly, including *clpks18* gene responsible for the synthesis of polyketide synthase enzyme (PKS) and *brn1* gene responsible for mechanical strength of appressorium required for penetration (Rižner and Wheeler, 2003). Trihydroxynaphthalene reductase (THR) is also an essential enzyme other than PKS of the DHN melanin biosynthesis pathway, and it represents an emerging target for the development of antimycotics. Secondary metabolite synthesis of this pathogen is regulated by genes involved in velvet protein biosynthesis (Gao et al., 2017), and the velvet-like B protein VelB plays a crucial role in controlling the production of conidia, cell wall composition, integrity, and host-specific methyl 5-(hydroxymethyl) furan-2-carboxylate toxin production. Studies have also reported involvement of VelB in pathogenicity as well as fungicide resistance in *C. lunata* (Wu et al., 2012; Gao et al., 2017).

The present study focuses on the identification of the potential virulence target of *C. lunata via* gene expression analysis, *in silico* approach, and *in vitro* evaluation of antifungal activity of natural bioactive compounds like α-pinene, eugenol, berberine, and curcumin.

## Material and methodology

### Sample collection and fungal isolation

Rice plant variety PUSA 1121 demonstrated typical symptoms of disease caused by *Curvularia* spp. including leaf lesions. The infected leaf samples were collected from Yamuna Nagar district, Haryana (GPS coordinates—30°02'38.1"N77°07'50.8"E). The collected samples were placed in sterile polythene bags properly tagged with date, time, and location (Naz et al., 2017). Sections of diseased leaf portions were surface-sterilized in 1% (w/v) sodium hypochlorite solution, rinsed in sterile distilled water, and incubated on fresh potato dextrose agar (PDA) for 96 h at 28 ± 2°C. Fungal isolates were identified macroscopically and microscopically (Sivanesan 1987; Cuervo-Parra et al., 2012) and further transferred to fresh PDA and incubated for 96 h at 28 ± 2°C.

### Molecular identification

Genomic DNA was extracted from *Curvularia* spp*.* using the modified cetyltrimethylammonium bromide (CTAB) method (Lee et al., 1988; Wu et al., 2001). Molecular identification of the isolate was confirmed by the amplification and sequencing of the full-length 18 S internal transcribed spacer (ITS) region using the ITS1 (5′-TCC GTA GGT GAA CCT GCGG-3′) and ITS4 (5′-TCC TCC GCT TAT TGA TATGC-3′) primers (White et al., 1990). The PCR-amplified ITS region was sequenced by Sanger sequencing. The sequences obtained were compared to the sequences in the GenBank database (www.ncbi.nlm.nih.gov.in) using basic local alignment search tool (BLAST) analysis, and identification was confirmed when 99–100% sequence identity was observed.

### Procurement of bioactive compounds

The compounds, namely, α-pinene, curcumin, berberine, and eugenol were procured from Sigma-Aldrich (India). The compounds were solubilised in dimethyl sulfoxide (DMSO) to make a stock solution of 100 mg/ml, except berberine (20 mg/ml in methanol). For working solution, stock solution was further diluted in potato dextrose broth (PDB). The final concentration of dimethyl sulfoxide (DMSO) never exceeded the amount with any detectable effect in assays (Szumilak et al., 2017).

### Antifungal susceptibility testing

The conidia were harvested in sterile phosphate-buffered saline (1× PBS), observed, and counted using a haematocytometer under a light microscope. The final conidial suspension was adjusted to 10^4^ conidia/mL in PDB (Amin and Abdalla, 1980; Xie et al., 2020). Minimum inhibitory concentration (MIC) of α-pinene, curcumin, berberine, and eugenol against *C. lunata* was determined using the broth microdilution method in a 96-well polystyrene plate according to CLSI protocol (CLSI, 2016; Alexander, 2017). Two-fold serial dilution was performed in a 96-well microplate to attain concentrations ranging from 5000 to 9.765 μg/ml. Each well was inoculated with 100 µL of the conidial suspension (as previously described in the section) except the negative control. The microplate was incubated at 28 ± 2°C for 5 days, and the growth in each well was compared with that of the positive control. The experiments were carried out in triplicate. The MIC value of a drug is determined as the lowest concentration with no visible growth relative to the drug-free control (Andrews, 2001).

### Pathogenicity test for *C. lunata* on onion peel epidermis

Onion bulb scales were thoroughly rinsed with distilled water. The inner epidermis of onion bulb scales was peeled off and cut into 1 × 1 cm^2^ strips. Sections of onion peel were floated on 4 ml distilled water in 60-mm Petri plates for treated and untreated samples. Freshly harvested conidia were washed with sterile water followed by centrifugation at 4500* g* for 10 min and resuspended in sterile distilled water to the final concentration of 1×10^4^ conidia/ml. Only conidial suspension (10 µl) was placed on a single strip as a positive control. MIC of α-pinene (1250 μg/ml), curcumin (78 μg/ml), berberine (156 μg/ml), and eugenol (156 μg/ml) was added with 10 µl of the conidial suspension on individual strips of onion peel epidermis. Each experiment was independently conducted in triplicate. The strips were incubated at 28 ± 2°C for 24 h. After 24 h of inoculation, extra suspension was removed from the peel, and 30% methanol was applied to prevent further penetration during observation (Chida and Sisler, 1987; Gupta et al., 2019). The strips were stained with lactophenol cotton blue and observed under a light microscope (×40 magnification) to observe hyphal growth and penetration. For scanning electron microscopy (SEM), the strips were sputter-coated with gold and observed under Zeiss SEM, MA EVO-18 Special Edition (Liu W. et al., 2011; Gupta et al., 2019).

### Biochemical assays

1) Melanin quantification: Isolation of melanin from *C. lunata* (treated and untreated) was performed by the modified method of Kumar et al. (2011). The fungus was cultured in the media supplemented with Inhibitory Concentration-50 (IC_50_) of α-pinene, curcumin, berberine, and eugenol in a 12-well cell culture plate. Inhibitory Concentration-50 (IC50) of α-pinene, curcumin, berberine, and eugenol were 625 μg/ml, 39 μg/ml, 78 μg/ml, and 78 μg/ml, respectively, where 50 % growth of *C. lunata* was inhibited. The extracted melanin was resuspended in 100 mM borate buffer, and absorbance was recorded in the wavelength range (250–800 nm) on a UV-visible spectrophotometer. Also, 100 mM borate buffer was used as a blank. The experiment was conducted in triplicate.
2) Conidial cell surface hydrophobicity (CSH) and conidiation: Using two-phase partitioning with hexadecane as the hydrocarbon phase, hydrophobicity assay was conducted (Pihet et al., 2009; Hoda et al., 2020). In brief, *C. lunata* conidia were harvested in 1× PBS from treated and untreated samples, and their absorbance was set to 0.3 using a spectrophotometer (at wavelength 630 nm). Hexadecane (500 µL) was added to conidial suspension and vortexed for 2 min at an interval of 30 s; then, for the hydrophobic phase, separation tubes were kept at room temperature for 10 min. At 630 nm, absorbance of the aqueous phase was determined and compared to the initial absorbance, that is, 0.30.

Percentage reduction in cell surface hydrophobicity (%CSH) was calculated for treated as well as untreated *C. lunata* conidia using the formula:

$$\%CSH=\frac{A1-A2}{A1}\times 100,$$

where A1 is the absorption before addition of hydrocarbon and A2 is the absorption after addition of hydrocarbon.

The effect of compounds on conidia formation was analysed by counting the number of spores using a haemocytometer (Abubakar and Likita 2021). Conidia were harvested from the 1 cm^3^ mycelial mat of treated and untreated cultures and resuspended in 1 ml of 1× PBS supplemented with 0.25% Tween-20. A volume of 100 µl of conidial suspension was placed on the surface of the counting chamber of the haemocytometer and covered with a cover slip. The number of conidia was counted from square grids in the counting unit of the haemocytometer. The conidia concentration was calculated.

### Gene expression analysis

The *brn1, velB, clm1, pks18,* and glyceraldehyde-3-phosphate dehydrogenase (*GAPDH*) gene sequences were downloaded from the NCBI (https://www.ncbi.nlm.nih.gov/pubmed) database for designing the primers for expression studies. The primers were designed by Primer3 software (http://primer3.ut.ee/) and were analysed for potential hair pin formation and self-complementarity (http://www.basic.northwestern.edu/biotools/oligocalc.html). The details of primers are given in Table 1.

**TABLE 1:** Gene-specific primers used for qRT-PCR.

S. No.	Gene name	Gene reference ID	Primer sequence (5′-3′)	Amplicon size (bp)
1	*velB*	KY435512.1	F: AGCATGGCTCACTACCAA	270 bp
			R: GTCCACCATGAGGACAAA	
2	*brn1*	JQ698339.1	F: AAC​AGC​CTT​TCA​ATC​CTC​TC	292 bp
			R: GTT​CAA​AGC​CTT​GAT​CTC​CT	
3	*clm1*	HQ851366.1	F: GGC​TAC​CAA​CAA​CCA​GAC​C	401 bp
			R: CTC​TGG​CCA​AAC​CAA​AAT​C	
4	*pks18*	MF114294.1	F- CGC​CAC​CTC​TGT​TCT​TCT​T	185 bp
			R- CCT​CAA​CAC​CAC​AAG​TCC​A	
5	*GAPDH*	LT715821.1	F- CATTGGCCGTATCGTCTT	339 bp
			R- GCCGTTGACAGTCAGGTT	

The expression of the genes of interest was quantified by quantitative real time-PCR (qRT-PCR) (Gupta et al., 2019, 2022). Mycelial cultures were harvested, and RNA was extracted using TRIzol™ reagent (Invitrogen). A measure of 2 μg of total RNA of each sample (treated and untreated) was used to synthesize first-strand cDNA by the oligo (dT)-18 primer using the Hi-cDNA Synthesis Kit (HiMedia). The qRT-PCR was performed using an ABI QuantStudio 3 system (Applied Biosystems, Streetsville, Canada), and amplification products were detected with SYBR Green Master Mix (G-Biosciences) for gene expression.

The relative quantification of individual gene expression was performed using the comparative threshold cycle method. The amplification program used for real time was 95°C for 3 min, 40 cycles at 95°C for 30 s, 60°C for 30 s, and 72°C for 30 s. To check the specificity of the PCR product, the melting curve was analysed at 95°C for 15 s, 60°C for 60 s, 72°C for 30 s, and holding stage 10 s. *GAPDH* gene was set as the reference gene. Relative expression was estimated using the 2^−ΔΔCt^ formula, where

$$\Delta \Delta Ct=\frac{\left[\left(Ct{\;}_{target\;gene}\right)\;sample-\left(Ct{\;}_{Tub}\right)\;sample\right]}{\left[\left(Ct{\;}_{target\;gene}\right)\;reference-\left(Ct{\;}_{Tub}\right)\;reference\right]}.$$

The results were analysed using ABI QuantStudio 3 software, and the genes were considered differentially expressed if they were at least two-fold up- or down-regulated.

### Molecular docking and dynamics studies

The three-dimensional structure of the proteins (VelB and THR) was not available in the Protein Data Bank (PDBhence FASTA sequence of virulence proteins velvet protein B (VelB) with accession number ARH19411 and 1,3,8-trihydroxynaphthalene reductase (THR) with accession number QTG11042—proteins of *C. lunata* were retrieved from the NCBI (https://www.ncbi.nlm.nih.gov/). To obtain the 3D structure of proteins, homology modelling was performed using SwissModel *via* the ExPaSy web server (https://swissmodel.expasy.org/). The Self-Optimized Prediction Method with Alignment (SOPMA) server was used to speculate the secondary structure of VelB and THR proteins (https://npsa-prabi.ibcp.fr/cgi-bin/npsa_automat.pl?page=/NPSA/npsa_sopma.html). The best template having maximum percentage identity with the target and modelled structure was then evaluated *via* PROCHECK. For model protein preparation such as charge assignment, solvation parameters, and fragmental volumes, Swiss-PdbViewer version 4.10 (SPBDV-4.10) was used (Morris et al., 2009). The 3D structures of the compounds (α-pinene, curcumin, berberine, and eugenol) were downloaded from the PubChem compound database (https://www.ncbi.nlm.nih.gov/pmc/articles/PMC4702940/) in the spatial data file (SDF) format. The PubChem compound identifier (CID) was 969516 (curcumin), 2353 (berberine), 3314 (eugenol), and 6654 (α-pinene). The SDF file format was further converted to the PDB file format using the Open Babel tool (https://openbabel.org/docs/dev/Installation/install.html) for molecular docking *via* the AutoDock4.2.3 tool (O’Boyle et al., 2011). The absorption, distribution, metabolism, excretion, and toxicity profiles of plant-derived compounds were qualitatively measured by using the online SwissADME program (http://www.swissadme.ch/index.php) (Sharma et al., 2022).

Molecular docking was performed using the AutoDock4.2.3 tool (Morris et al., 2009; Karthika et al., 2021) to predict the binding and the structure of the intermolecular complex between drug targets and potential inhibitors. The Lamarckian genetic algorithm was utilized for protein–ligand interactions with the set parameters. The total number of poses was set to 50. Poses were further clustered using all atom root mean square deviation (RMSD) cut-off of 0.3 Å to remove redundancy. The default values were used for all other parameters for docking and scoring. The protein structure was kept rigid in all steps. The molecular interactions of the best docking pose were visualised *via* Discovery Studio Visualizer programs (http://accelrys.com/products/collaborative-science/biovia-discovery-studio/visualization-download.php). The amino acid residues that displayed interactions with the ligand are documented in Table 2 and Table 3.

**TABLE 2:** Molecular docking affinity of four compounds with THR protein of *C. lunata*.

Compound name	PubChem CID	THR protein		
		Binding affinity (Kcal/mol)	Hydrogen bond formation	Amino acid residues
Curcumin	969516	-10.80	4	TYR178, ILE41, ASN114, and ARG39
Berberine	2353	-9.62	3	ILE41 and SER164
Eugenol	3314	-6.25	3	ILE165, SER164, and PRO208
α-Pinene	6654	-6.13	0	-

**TABLE 3:** Molecular docking affinity of four compounds with VelB protein of *C. lunata*.

Compound name	PubChem CID	VelB protein		
		Binding affinity (Kcal/mol)	Hydrogen bond formation	Amino acid residues
Curcumin	969516	-8.03	4	SER124, SER119, and SER132
Berberine	2353	-6.98	0	-
Eugenol	3314	-5.21	1	GLU130
α-Pinene	6654	-4.39	0	-

All-atom molecular dynamics (MD) simulation was performed to understand the conformational stability of VelB and THR proteins bound with the docked molecules in comparison to the unbound state of proteins. Atomic coordinates of VelB and THR protein complexes with docked molecules were used to generate the simulation trajectory using GROMACS v5.1.4 (Abraham et al., 2015). During the simulation complex preparation stage, the CHARMM27 (Bjelkmar et al., 2010) force field was used for proteins, while the TIP3P water model was used to solvate the protein complex. The bound ligand parameters were generated as described in the literature (Zoete et al., 2011). The protein complex was placed in the centre of the cubical box with 10 Å edge-side filled with water molecules. The total charge of the simulation box was neutralised by adding 0.15 M counterions (Na^+^Cl^−^). All the MD simulations were performed under physiological conditions (Joung and Cheatham, 2008). The prepared simulation box was taken for energy minimization using steepest descent followed by conjugant gradients (50,000 steps for each). The system was further equilibrated through the constant number, volume, and temperature (NVT) and the constant number, pressure, and temperature (NPT) for 500 ps. The Berendsen thermostat (Berendsen et al., 1987) and the Parrinello–Rahman pressure (Parrinello and Rahman, 1980) algorithm were used to maintain the temperature and pressure, respectively. Final MD simulation was performed for 100 ns under the NPT ensemble condition with the step size of 0.2 fs. GROMACS modules and MD trajectory (McGibbon et al., 2015; Sankar et al., 2021) were employed to visualize the global structural order parameters: RMSD, radius of gyration (RoG), solvent-accessible surface area (SASA), and root mean square fluctuation (RMSF).

### Statistical analysis

Statistical analysis was performed using the one-way analysis of variance (ANOVA) for the comparison of results of gene expression analysis *via* qRT-PCR and melanin and CSH percentage for biochemical assays. The experiment was conducted in technical and biological triplicate. Statistical analysis was also performed using GraphPad Prism software 8.0.2.263 version and Microsoft Excel. *p* < 0.05 was considered statistically significant.

## Results

**FIGURE 1:**
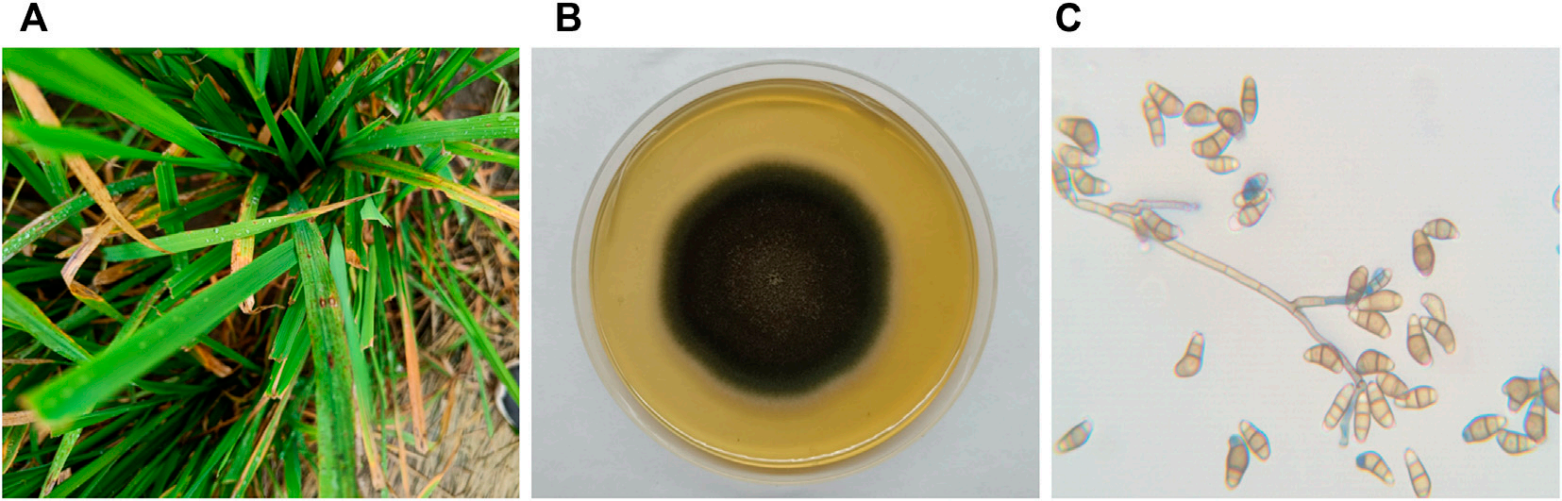
**(A)** *C. lunata* infection on the leaves of the rice plant; **(B)** *C. lunata* colony morphology on potato dextrose agar; and **(C)** *C. lunata* conidia.

Rice plants with symptoms of blast disease specifically leaf lesions were collected from Yamuna Nagar, Haryana, India (Figure 1). The fungal isolate was identified as *Curvularia* based on its colony morphology, viz., fluffy and velvety mycelia, greyish black colour, black pigment on the reverse side, and straight to pyriform conidia having three to four cells, with a large and curved central cell with smooth-walled in a lactophenol cotton blue mount. Morphological characteristics of the isolated fungus were in agreement with Ellis (1971) and Sivanesan (1984).

Furthermore, molecular characterisation and identification of the fungal isolate at the species level were conducted and confirmed by the amplification and sequencing of 18 S ITS1 and ITS4 regions. The obtained sequence showed 100% similarity with *C. lunata* from the GenBank database*.* The sequence was submitted to the NCBI with GenBank accession number OL757869 (https://www.ncbi.nlm.nih.gov/search/all/?term=OL757869).

### Antifungal susceptibility testing

The isolated fungal pathogen was susceptible to polyenes and azoles. The calculated MIC of bioactive compounds against *C. lunata* was in the range of 1250–78 μg/ml: curcumin (78 μg/ml), berberine (156 μg/ml), eugenol (156 μg/ml), and α-pinene (1250 μg/ml). Among all, curcumin inhibited the pathogen hyphal and conidial germination at a very low concentration. At IC_50_ of α-pinene, white fungal morphology was observed *in vitro*, whereas IC_50_ of other compounds inhibited the growth of fungi to 50% with minor changes in its morphology. IC_50_ of the compound referred when the growth of microorganism was suppressed by 50%.

### Pathogenicity test of *C. lunata* conidia on onion peel epidermis

**FIGURE 2:**
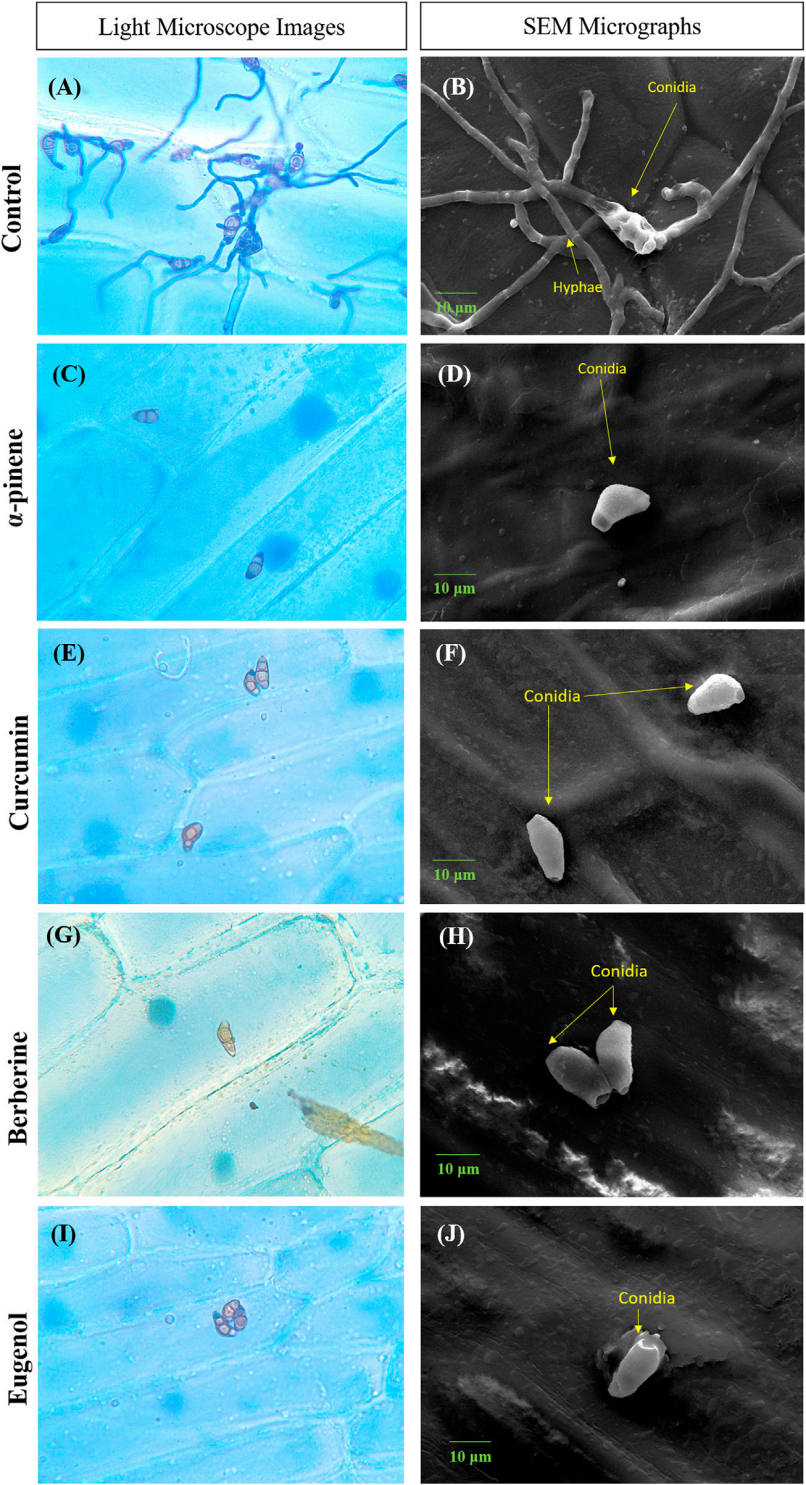
Microscopic images depicting conidia of *C.lunata* Control **(A,B)**; α-pinene **(C,D)**; curcumin **(E,F)**; berberine **(G,H)**; and eugenol **(I,J)**. Scale bar = 10 µm.

Conidia failed to adhere to onion peel *epidermis*, in the presence of bioactive compounds when observed after 24 h of incubation. No visible conidial germination was observed at MIC of α-pinene, curcumin, berberine, and eugenol. In the control sample, conidia with dense hyphal growth were observed on the onion peel under a light microscope as well as SEM as depicted in Figure 2. Upon treatment, the shape of conidia was obovoidal to clavate, curved at subterminal ends, and treatment with bioactive compounds effectively prevented the germination of conidia.

### Biochemical assays

1) Melanin content: The overall characteristic absorption of melanin was observed at 205 nm which was 0.604, 0.401 0.274, 0.247, and 0.184 for control, berberine, eugenol, α-pinene, and curcumin, respectively (Supplementary Figure S1; p < 0.0001). The melanin content in the compound-treated *C. lunata* culture showed significant reduction in the quantity as compared to that of control.
2) CSH percentage: A statistical decrease was observed in the biochemical CSH value of treated cultures as compared to the untreated positive control. The calculated CSH percentage was 87.05%, 79.57%, 75.54%, and 58.73% for α-pinene-, eugenol-, berberine-, and curcumin-treated *C. lunata* as compared to the control (Supplementary Figure S2; p < 0.05). Curcumin affected the hydrophobicity of conidia more than the other compounds.
3) Culture was grown at IC_50_ of compounds to check the number of conidia formed, and a 50% reduction in conidiation was observed in treated cultures in comparison with the control.

### Gene expression analysis

**FIGURE 3:**
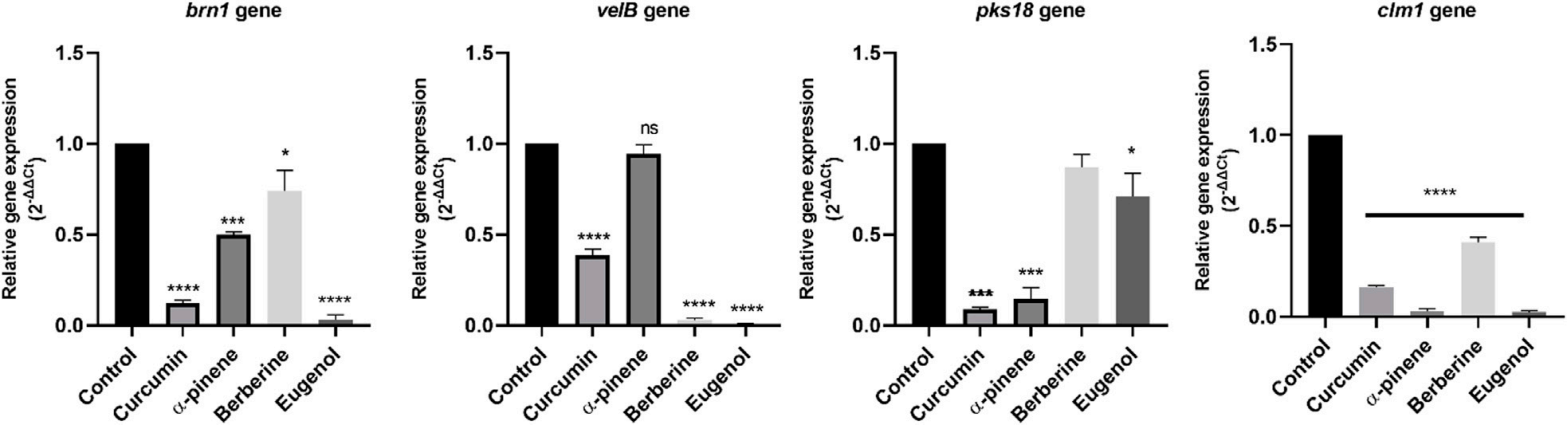
Relative quantification of *brn1*, *velB*, *pks18*, and *clm1* gene expression in *C. lunata* (normalised to the house-keeping gene GAPDH). Data were reported as mean of fold changes with standard deviation from two independent experiments amplified in triplicate. *p* ≤ 0.05 was considered statistically significant.

The effect of curcumin, α-pinene, berberine, and eugenol treatment on the expression of *brn1*, *velB*, *pks18*, and *clm1* of *C. lunata* was investigated by reverse transcription followed by qRT-PCR for differential gene expression. α-pinene, curcumin, berberine, and eugenol treatment led to a significant down-regulation of *velB*, *brn1*, *clm1*, and *pks18* gene transcripts in comparison with the control (untreated) (Figure 3). The expression of secondary metabolite gene *brn1* was significantly down-regulated upon eugenol treatment, followed by curcumin, α-pinene, and berberine and *pks18* genes in curcumin and α-pinene. The *velB* gene was significantly down-regulated in eugenol, berberine, and curcumin. The relative expression of *clm1* gene was highly down-regulated in all treated samples as compared to the control. The complete expression data were normalized by the housekeeping gene *GAPDH*. Gene expression data expressed as 2^−ΔΔCt^ are the mean of at least three replicates ± standard error.

### Molecular docking studies and dynamics studies

**FIGURE 4:**
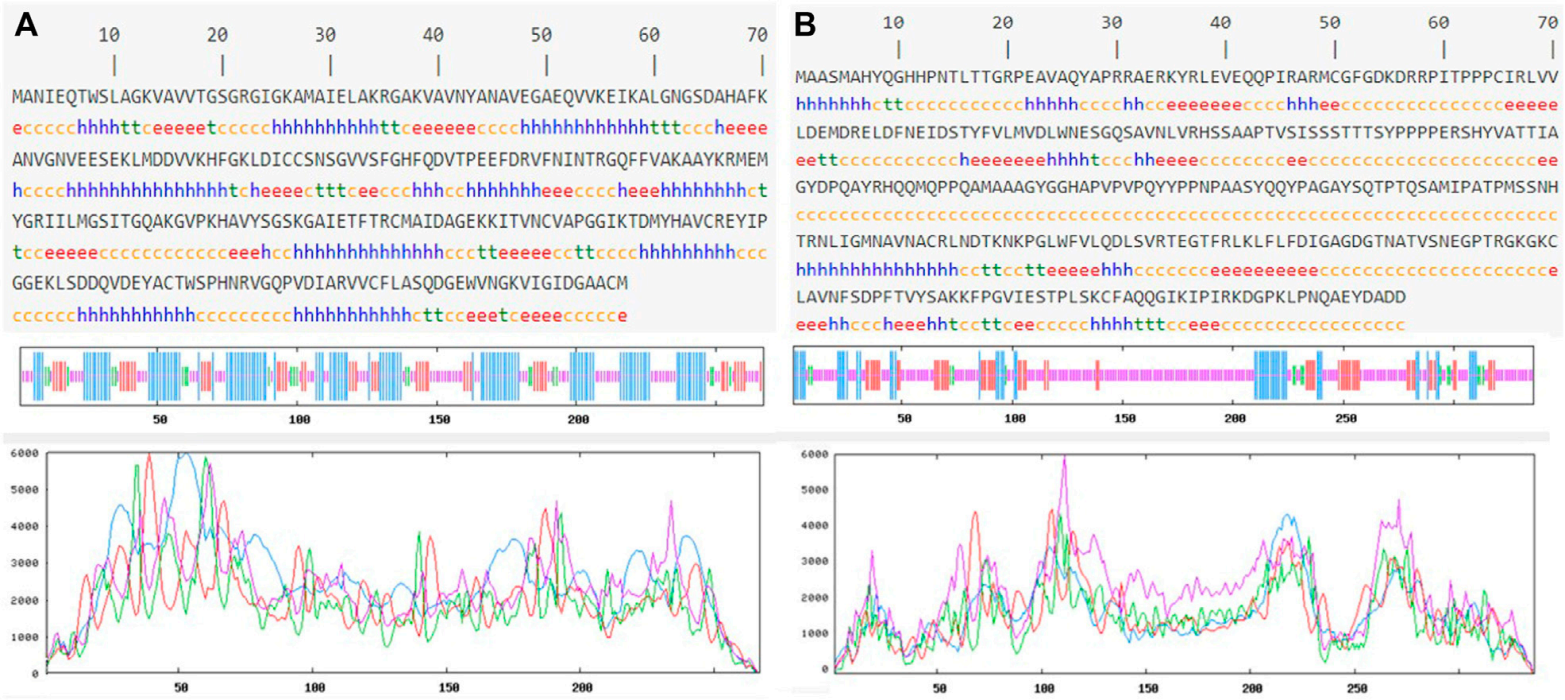
Predicted secondary structure validation of THR **(A)** and VelB **(B)** proteins of *C. lunata* using SOPMA. The blue line represents α-helices, red colour represents the extended strand, green colour represents β-turn, and magenta colour represents the random coil in graphical representation. The *X*-axis represents position of the amino acid; the *Y*-axis shows the score for each predicted state.

Prediction of the secondary structure of THR (Figure 4A) resulted in 40.82% α-helix (h), 18.35% extended strand (e), 7.87% β-turn (t), and 32.96% random coil (c) elements, and the VelB sequence (Figure 4B) consisted of 15.18% α-helix (h), 17.26% extended strand (e), 4.46% β-turn (t), and 63.10% random coil (c) elements. Graphs were obtained to visualize the prediction and score curves for all predicted states using parameters such as window width and number of states.

**FIGURE 5:**
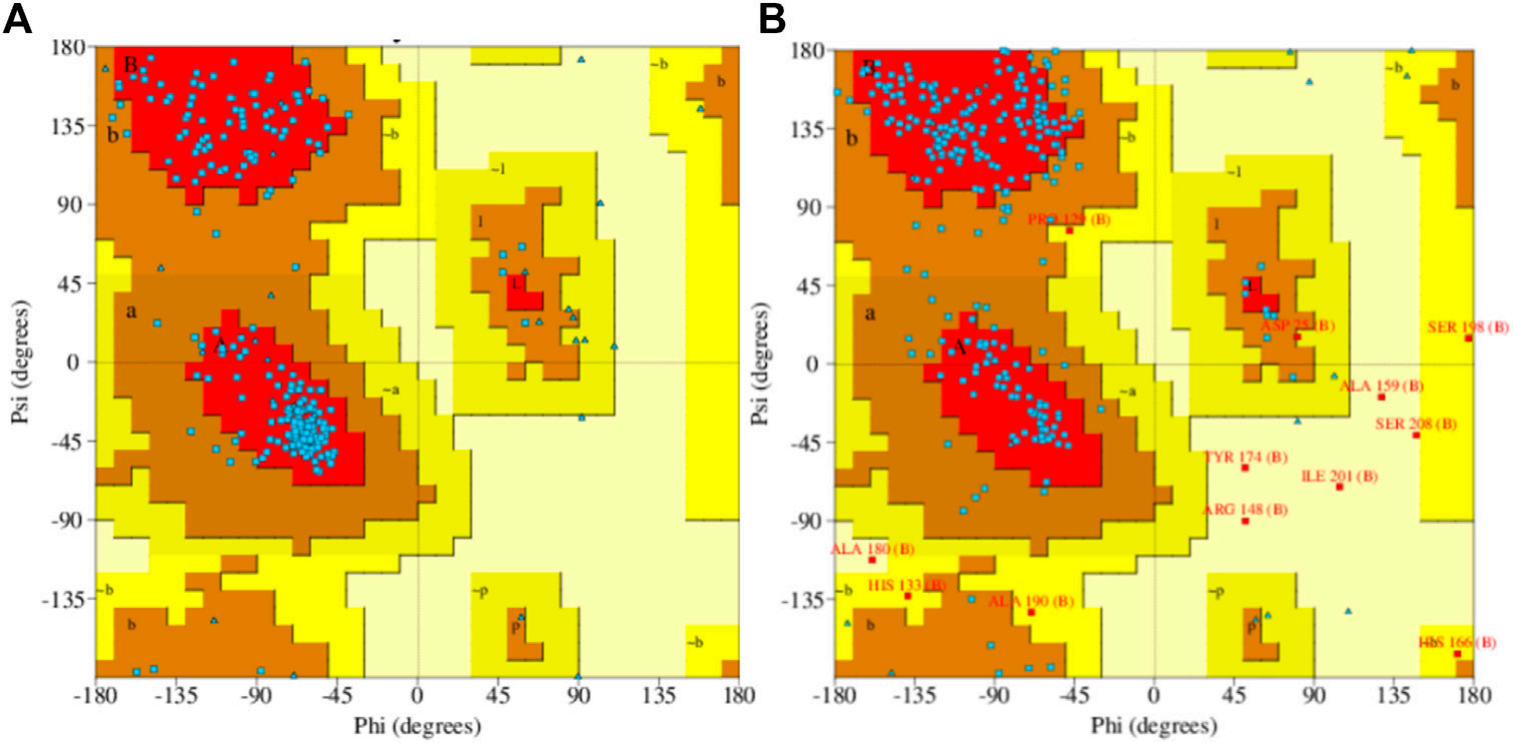
Ramachandran plot of predicted 3D structures of THR **(A)** and VelB **(B)** proteins of *C. lunata* using PROCHECK software.

Model accuracy assessment of modelled protein structures of THR and VelB of *C. lunata* was performed *via* SwissModel. The PDB ID 1YBV was used as the template to model the 3D protein structure of the THR protein sequence and PDB ID 4N6R chain B for VelB protein. The stereochemical quality and accuracy of the model were tested using PROCHECK. Results from PROCHECK were reported as the Ramachandran plot. For THR protein, 89.0% residues were in most favoured regions, 11.0% residues were in additional allowed regions, and no residues were in disallowed regions. Similarly, in protein VelB, 75.7% residues were in most favoured regions, 19.8% residues were in additional allowed regions, 2.1% residues were in generously allowed regions, and 2.5% residues were in disallowed regions. Both modelled proteins obtained by homology modelling were of good quality on the basis of the Ramachandran plot (Figure 5).

**FIGURE 6:**
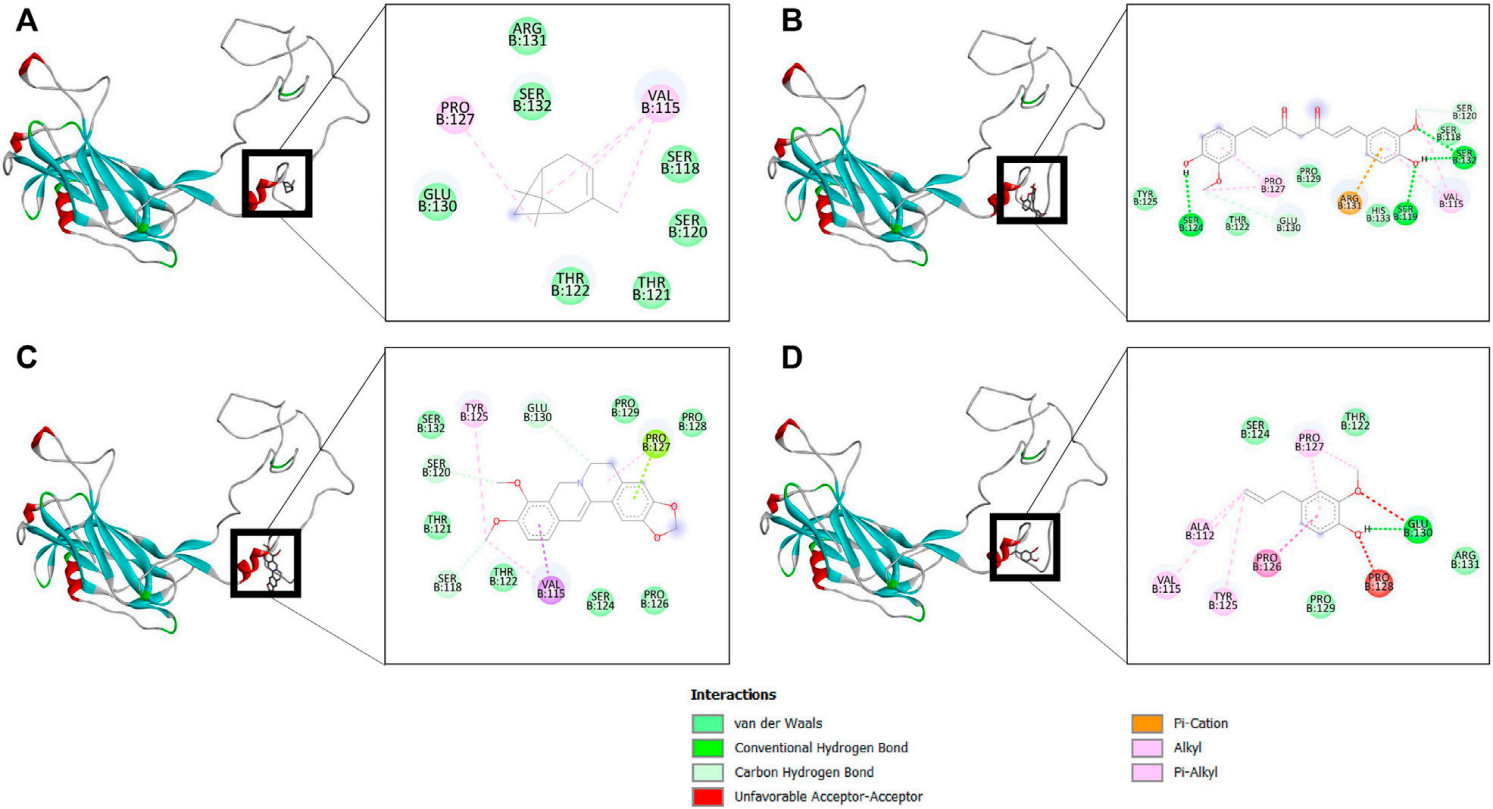
Binding of **(A)** α-pinene, **(B)** curcumin, **(C)** berberine, and **(D)** eugenol with VelB. Ribbon and 2D representation of VelB protein showing various interactions and docking fit of compounds.

**FIGURE 7:**
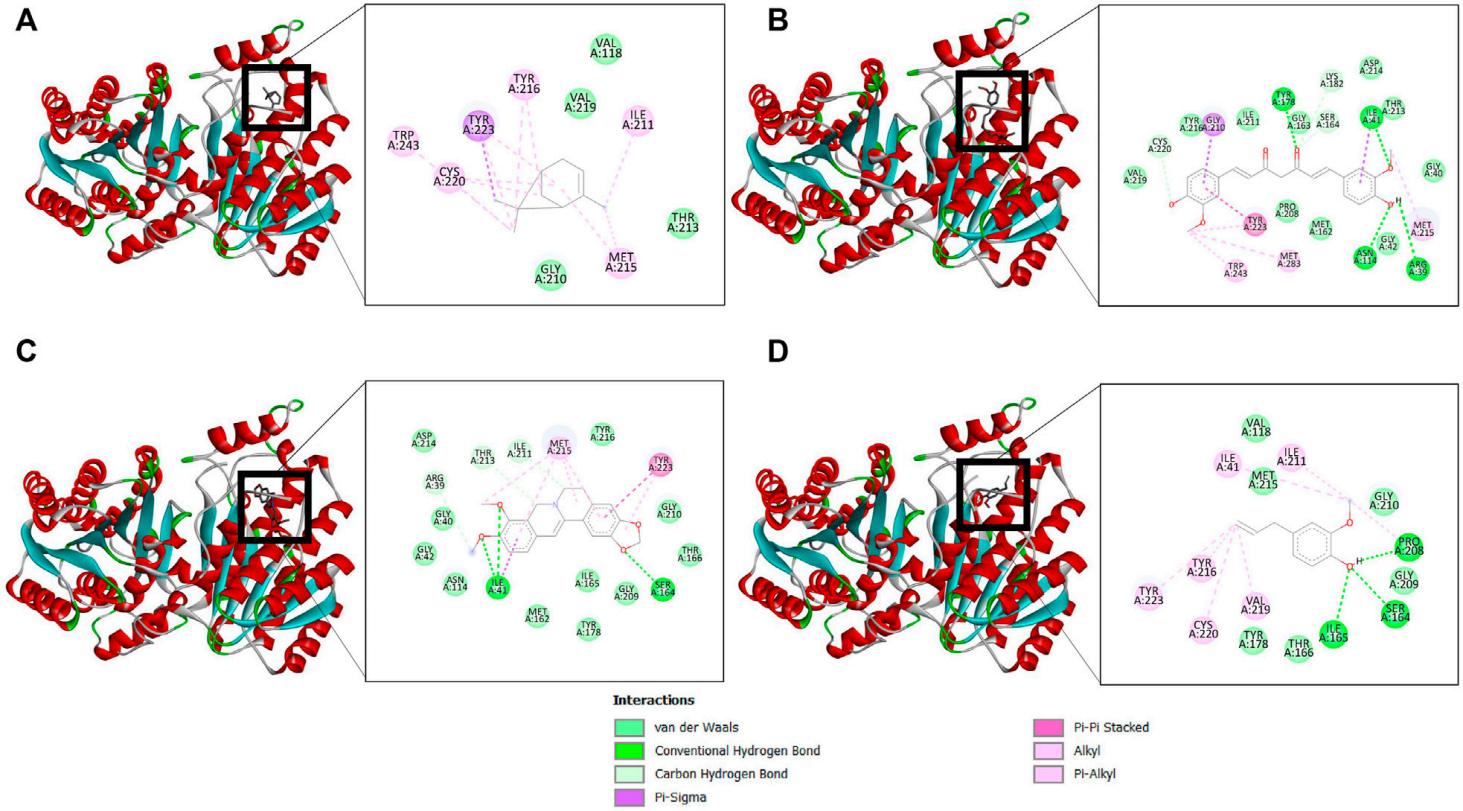
Binding of **(A)** α-pinene, **(B)** curcumin, **(C)** berberine, and **(D)** eugenol with THR. Ribbon and 2D representation of THR protein showing various interactions and docking fit of compounds.

The binding affinity of four natural compounds with THR and VelB proteins of *C. lunata* are presented in Tables 2 and 3, respectively. The binding interaction of four natural compounds with VelB and THR proteins are shown in Figures 6, 7, respectively. The binding affinity and hydrogen bonds of curcumin were high with THR protein followed by berberine, eugenol, and α-pinene. With VelB protein, curcumin showed the highest binding affinity and hydrogen bonds as compared to berberine, eugenol, and α-pinene. The binding affinity of curcumin was -10.80 Kcal/mol with four hydrogen bonds for THR protein and −8.03 Kcal/mol with four hydrogen bonds for VelB protein. Berberine also showed good binding affinity for THR and VelB proteins, i.e., −9.62 Kcal/mol and −6.98 Kcal/mol, respectively. Berberine did not form any hydrogen bonds with VelB protein. Eugenol interacted with THR protein with three hydrogen bonds, while only one hydrogen bond was formed with VelB protein. α-Pinene showed binding affinity −6.13 Kcal/mol and −4.39 Kcal/mol with THR and VelB proteins respectively, but there was no hydrogen bond formation.

Drug-likeness properties of α-pinene, curcumin, berberine, and eugenol were evaluated *via in silico* ADME-Tox analysis (Table 4). All four compounds followed Lipinski’s Rule of Five without any violation and therefore could be administered as oral drugs. The pharmacokinetics drug properties for compounds α-pinene, curcumin, berberine, and eugenol resulted good drug ability characteristics which included molecular weight <500 g/mol, lipophilicity (MlogP) < 5, hydrogen bond acceptor <5, and hydrogen bond donor <10.

**TABLE 4:** Physicochemical analysis of potential inhibitors of THR and VelB proteins of *C. lunata*.

Compounds	ADME properties (Lipinski’s Rule of Five)	Molecular weight	Molecular formula	Radar diagram
α-Pinene	Molecular weight (g/mol)	136.23	C_10_H_16_	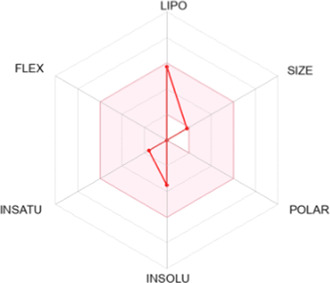
	LogP	3.44		
	H-bond donor	0		
	H-bond acceptor	0		
	Violation	0		
Curcumin	Molecular weight (g/mol)	368.38	C_21_H_20_O_6_	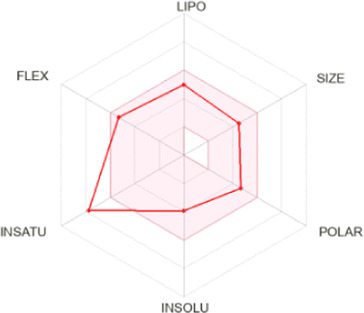
	LogP	1.47		
	H-bond donor	2		
	H-bond acceptor	6		
	Violation	0		
Berberine	Molecular weight (g/mol)	336.36	C_20_H_18_NO_4_	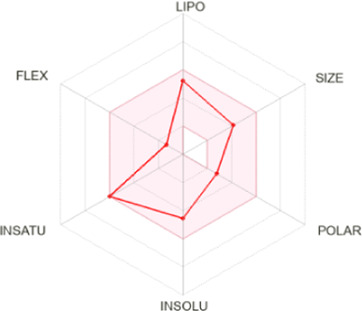
	LogP	2.19		
	H-bond donor	0		
	H-bond acceptor	4		
	Violation	0		
Eugenol	Molecular weight (g/mol)	164.20	C_10_H_12_O_2_	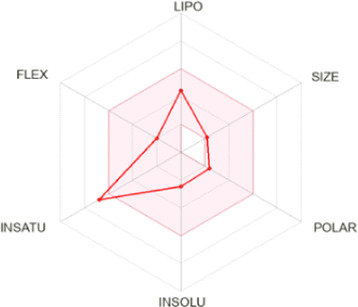
	LogP	2.25		
	H-bond donor	1		
	H-bond acceptor	2		
	Violation	0		

Note: According to Lipinski’s rule, molecular weight <500 g/mol, lipophilicity (MlogP) <5, hydrogen bond acceptor <5, and hydrogen bond donor <10 are considered.

**FIGURE 8:**
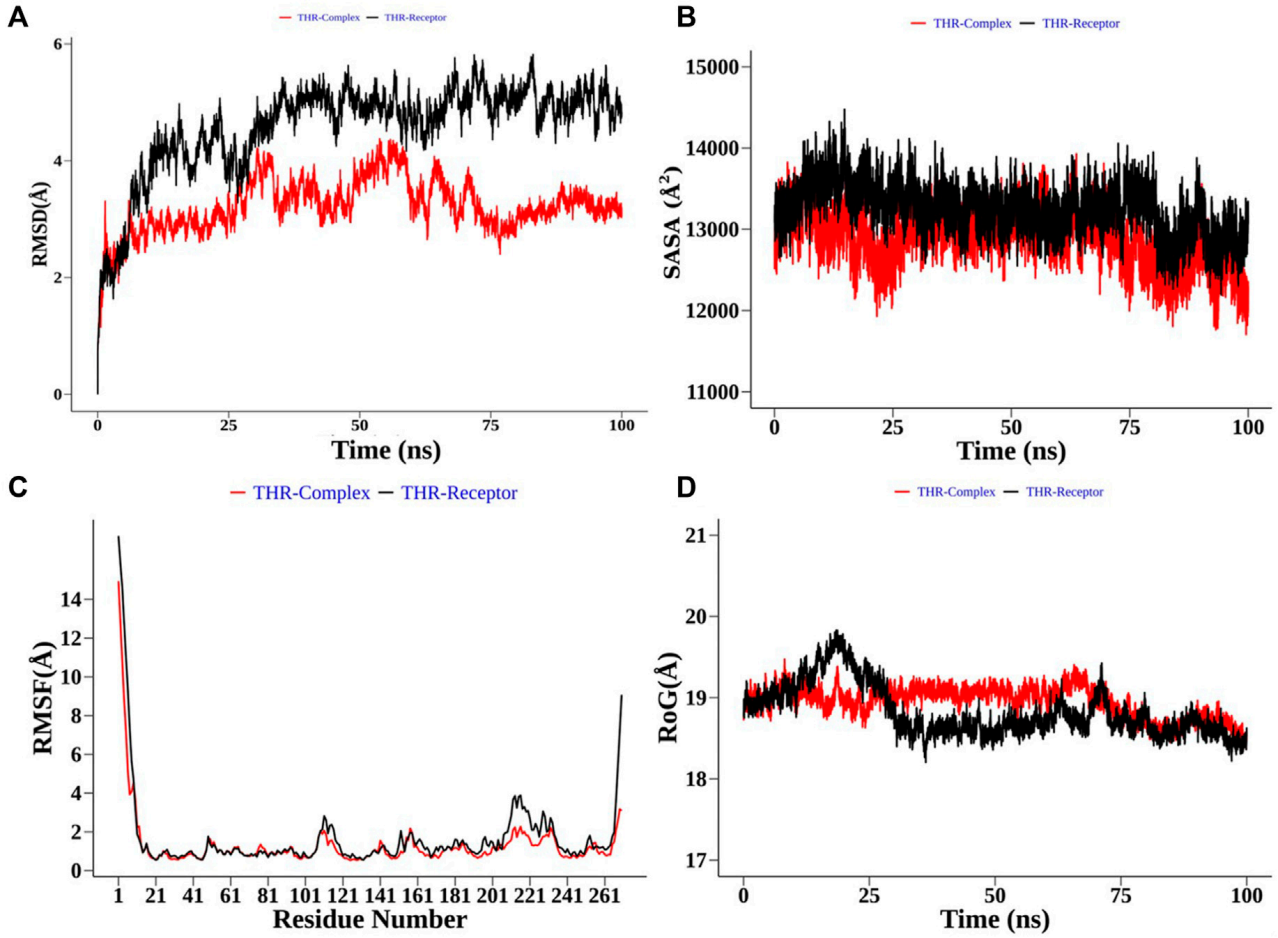
Structural order parameter analysis of the THR complex (curcumin) with respect to the THR receptor. **(A)** Root mean square deviation (RMSD), **(B)** solvent-accessible surface area (SASA), **(C)** root mean square fluctuation (RMSF), and **(D)** radius of gyration (RoG) analysis.

Structural order parameters of the THR complex were analysed with respect to the THR receptor to depict the structural changes upon curcumin binding. The RMSD distribution (Figure 8A) reflected that the inhibitor bound state of THR experiences comparatively less structural deviation along the simulation time and has 3.22 Å mean RMSD, while the THR receptor has 4.58 Å mean RMSD. RoG defines the compactness of the structure, and Figure 8D shows that RoG remained consistent for the THR complex in comparison to the THR receptor which further highlights that curcumin binding at the active site of THR protein enhances the structural stability. The same was confirmed by SASA analysis (Figure 8B). Apart from the protein structure-level dynamics, the protein residue-level dynamics was attained by RMSF analysis (Figure 8C) which depicted that in the presence of an inhibitor, binding site residues 114 and 200–220 have less fluctuation and therefore stabilize the complex.

**FIGURE 9:**
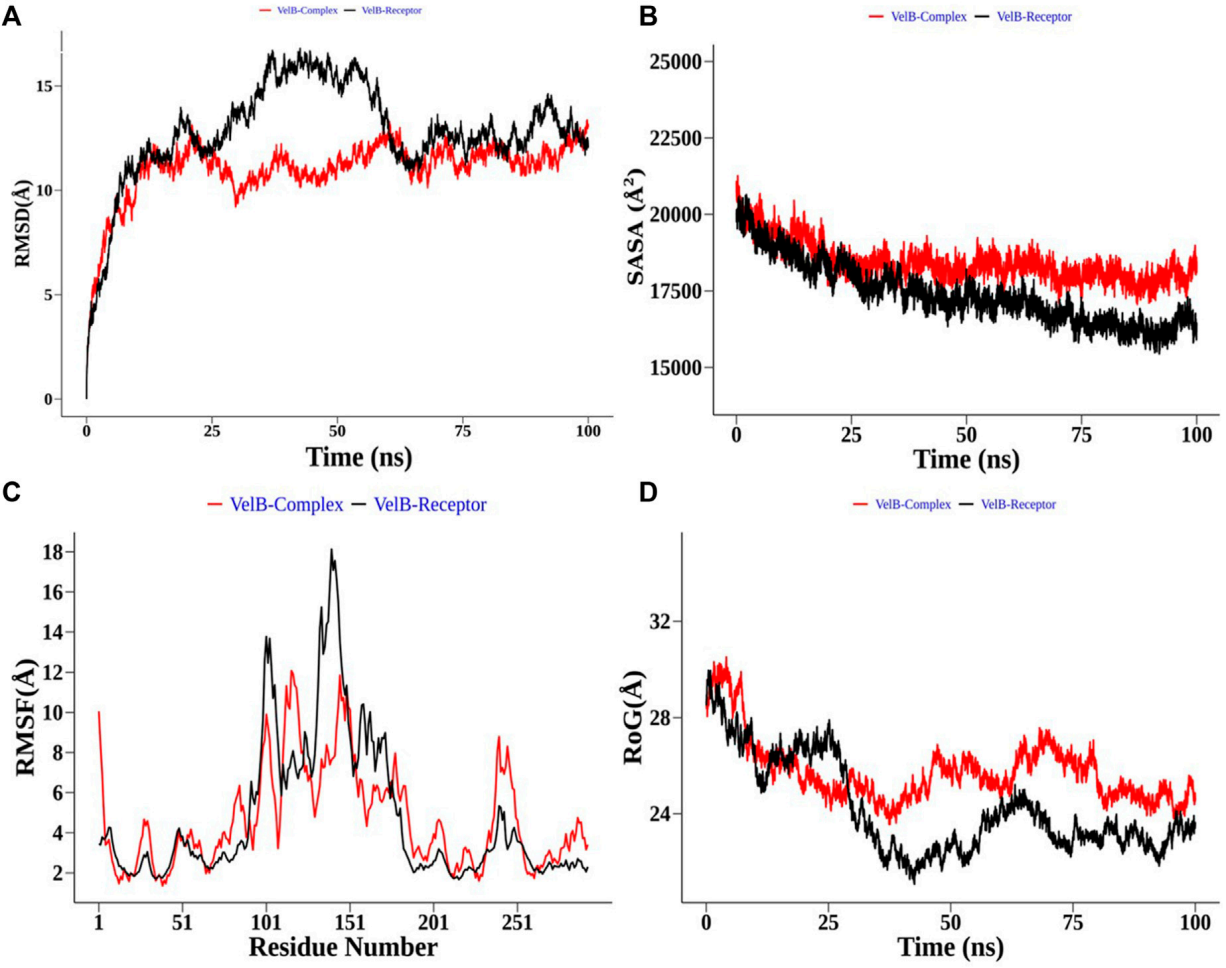
Structural order parameter analysis of the VelB complex (curcumin) with respect to the VelB receptor. **(A)** Root mean square deviation (RMSD), **(B)** solvent-accessible surface area (SASA), **(C)** root mean square fluctuation (RMSF), and **(D)** radius of gyration (RoG) analysis.

In the case of the VelB complex, structural order parameter analysis with respect to the VelB receptor showed that RMSD distribution showed a more zigzag pattern for receptor protein with the mean RMSD value of 12.81 Å, while the bound complex attained more stability in the presence of an inhibitor (Figure 9A). RoG (Figure 9D) and SASA analyses (Figure 9B) further explained the stability of the bound complex along the simulation. SASA achieved the plateau after 25 ns, while SASA for the unbound state receptor was still declining. Residue-level RMSF analysis also displayed that binding site residues 110–135 restricted the local fluctuation, thereby increasing the stability of the complex (Figure 9C). Curcumin binding stability at the binding site of respective proteins was further computed and analysed by monitoring the average distance between centre mean position of curcumin and selected binding site residues as shown in Supplementary Figure S3 for the THR–curcumin complex and Supplementary Figure S4 for the VelB–curcumin complex. In case of the THR complex, binding site residues such as ARG39, ASN114, SER164, TYR178, and LYS182 retained the equilibrium distance after 25 ns. As ASN114, TYR178, and LYS182 were present at the beta and helix secondary structure, the distances from these residues were found to be more consistent compared to ARG39 and SER164 which were located in the turn of the secondary structure. This analysis showed that binding site residues maintained favourable distance for a stable molecular interaction. In case of the VelB complex, all binding site residues were located at the coiled coil secondary structure, and analysis of the centre mean distance from curcumin with binding site residues will further provide evidence of curcumin stability in the coiled coil binding region of VelB receptor protein. As it can be seen from Supplementary Figure S4, all six selected binding site residues (ARG131, PRO127, SER119, HIS133, SER118, and TYR125) maintained equilibrium distances after 25 ns simulation time. All distances have undergone shape drifts transiently at ∼65 ns and thereafter returned to initial equilibrium distances. This calculation clearly suggests that despite the coiled coil region of the binding site, curcumin well oriented in the VelB-binding site. To further establish the fact that curcumin orientation in the protein-binding site of both proteins remains stable, curcumin RMSD was measured and plotted in a time evolution manner in Supplementary Figure S5. Similar to the distance in Supplementary Figures S3, S4, curcumin stabilized its orientation in the respective binding site after 25 ns of simulation.

**FIGURE 10:**
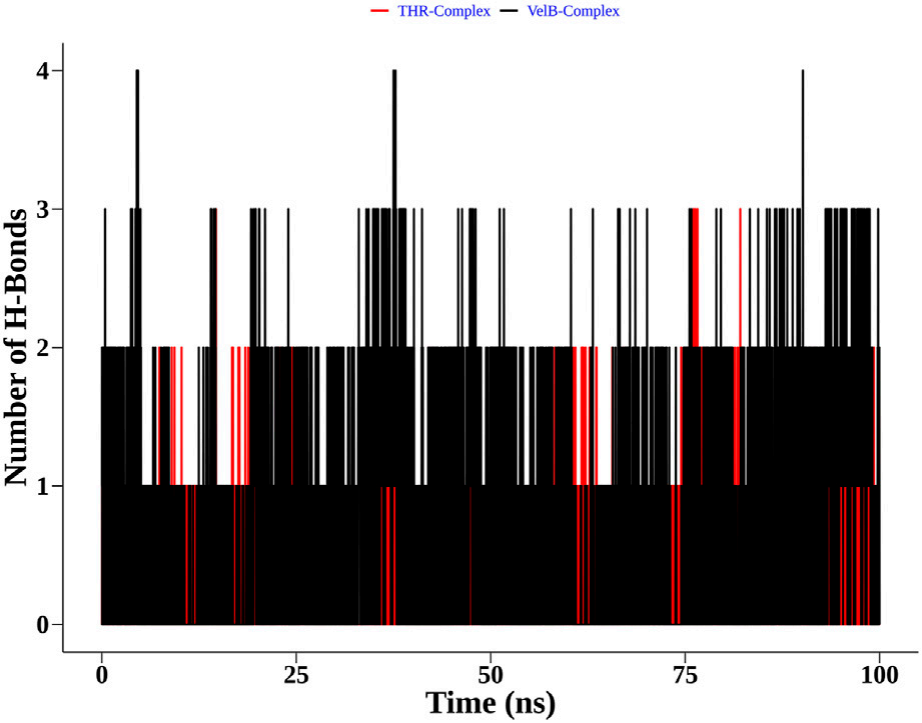
Distribution of the hydrogen bonds formed by curcumin with THR and VelB receptors at the binding site.

Apart from complex structural stability, interaction stability was assessed by calculating the number of hydrogen bonds formed between curcumin and receptor protein along the simulation time. The THR–curcumin complex formed on average one hydrogen bond with more than 90% occupancy, while the VelB–curcumin complex formed on average one hydrogen bond with more than 82% of simulation time (Figure 10).

## Discussion

A fundamental problem with *Curvularia* infections is the risk of transferring a genetically evolved isolate from farms to humans. Such transferability through the human–plant interaction or intake of contaminated air could cause resistance against available antifungal drugs since field isolates suffer from fungicide selective pressure and undergo virulence differentiation to adapt to adverse conditions (Bengyella et al., 2017)*. Curvularia* spp. are sensitive to triazoles, and there are chances of interlocking lifestyle and fungicide pressure which may lead to the development of resistance in immunocompromised patients receiving azole therapy (Bengyella et al., 2017). Occurrence of antifungal resistance in fungal diseases along with a genetically evolved *Curvularia* spp. imparts the search for new therapy to control fungal infections though it is a major challenge of present-day treatment. There are known natural compounds that can control the disease caused by *C. lunata*; for example, essential oil of *Cymbopogon citratus* and extract of *Cinnamomum zeylani* are reported to inhibit conidial germination and reduce disease progress (Mishra et al., 2009; Mourão et al., 2017).

In the present study, natural compounds, namely, α-pinene, curcumin, berberine, and eugenol were studied for their antifungal activity against *C. lunata*. The fungal growth was completely inhibited by curcumin (78 μg/ml), berberine (156 μg/ml), eugenol, (156 μg/ml) and α-pinene (1250 μg/ml). In the studies conducted elsewhere, α-pinene showed significant antifungal activity, with greater inhibitory activity against *Candida parapsilosis*, and proved to be effective in inhibiting fungal growth (Nóbrega et al., 2021). Previously reported MIC of berberine was 125 μg/ml against *C. lunata* (Kokkrua et al., 2020), whereas in the current study it was calculated as 156 μg/ml. Eugenol also inhibited the growth of fungi at 156 μg/ml, and similar results were reported for the antifungal activity of eugenol against *T*. *rubrum* strains (64–512 μg/ml) (de Oliveira Pereira et al., 2013). The extract of *Curcuma longa* also reported strong inhibitory effect on various pathogenic fungi at 1 mg/ml concentration (Chen et al., 2018), and antifungal effect of curcumin has been reported on *Aspergillus* spp. at 0.2 mg/ml (Martins et al., 2008; Gitika et al., 2019) and *Candida* spp. at 0.1–2 mg/ml (Narayanan et al., 2020).

The interaction between host plants and conidia of *C. lunata* begins with the adherence of conidia onto the leaf surface (Xie et al., 2020). Once the conidia adhere, they start to germinate and form appressoria. The cell wall of appressoria contains melanin which aids to provide mechanical strength for host tissue penetration. In *C. lunata*, sporulation and germination are crucial steps for spreading the disease (Xie et al., 2020). In the present study, our compounds suppressed sporulation as well as hyphal growth at IC_50_ (curcumin, 39 μg/ml; berberine, 78 μg/ml; eugenol, 78 μg/ml; and α-pinene, 625 μg/ml). The spore germination and its pathogenicity were studied on the onion epidermis layer, where the compound-treated (α-pinene, curcumin, berberine, and eugenol) conidia failed to adhere and germinate on the surface. The invasive hyphae of *C. lunata* were observed when untreated conidia were observed under light and electron microscope. Another study on spore germination of *Curvularia maculans* reported that berberine affected the germination process of the fungus (Basha et al., 2002). Biochemical assay showed that curcumin exhibited a significant reduction in conidial hydrophobicity as compared to the control. It might be due to curcumin interacting with the conidial wall/membrane and disturbing the cell integrity (Srivastava et al., 2020).

Unlike other fungi*,* not much is studied about *C. lunata* pathogenesis at the molecular level. Melanin is one of the reported virulence factors which can improve mechanical strength of appressorium required for penetration inside the host (Rižner and Wheeler, 2003). It is produced from 1,8- dihydroxynaphthalene (DHN) *via* the pentaketide pathway in this fungus; crucial genes involved in the pathway are *pks18*, *scd1*, and *brn1* (Baker et al., 2006; Liu T. et al., 2011). According to Liu T. et al (2011) and Wang et al. (2020), *brn1* gene deletion led to improper DHN-melanin biosynthesis as well as accumulation in the cell wall which also affects the production of other mycotoxins by this fungus. The current study observed significant down-regulation of *pks18* and *brn1* genes when the pathogen comes in contact with bioactive compounds. The velvet genes/proteins also play a significant role in regulating secondary metabolism, cell wall integrity pathway, and sporulation (Calvo, 2008; Gao et al., 2017). It has been reported that deletion of *velB* gene exhibited reduced growth rate and conidiation with increased aerial hyphae formation (Gao et al., 2017). In the present study, the expression of *velB* gene, which is a key member of velvet protein synthesis, was significantly down-regulated in the presence of α-pinene, curcumin, berberine, and eugenol. Mitogen-activated protein kinase gene *clm1* regulated cell wall integrity, conidiophore formation, and cell-degrading enzyme activity (Liu T. et al., 2011; Ni et al., 2018). The presence of bioactive compounds (α-pinene, curcumin, berberine, and eugenol) showed that there was a significant down-regulation of *clm1* gene of *C. lunata*, which might result in decreased cell wall integrity as well as conidia formation. The qRT-PCR analysis showed that the expression level of *pks18*, *brn1*, *velB*, and *clm1* genes were reduced compared to that in the control. Hence, conidia were unable to maintain integrity for their growth and sporulation.

Reported mutant studies of *brn1*, MAPK gene, and toxin-related gene deletion led to a decrease in sporulation as well as virulence (Liu T. et al., 2011; Gao et al., 2012). In case of compound treatment, it was found that the conidia germination process was inhibited as shown in SEM images, and therefore the expression of *brn1*, *clm1*, and *velB* genes could be decreased. Another mutant study reported that *ΔClVelB* showed high expression of *pks18*, *brn1*, and *cmr1* genes at 48 h and 60 h gene expression study (Gao et al., 2017). Also, *velB* gene deletion indicated the increase in osmotic resistance which suggests that *velB* gene involved in the regulation of cell wall integrity. Our study also suggested that the presence of compounds decreases *velB* gene expression which might result in decreased toxin production and cell wall integrity.

Furthermore, to understand the possible targets of compounds (α-pinene, curcumin, berberine, and eugenol), an *in silico* docking approach was used for virulence proteins involved in the melanin pathway and conidiation. The virulence proteins THR and VelB were responsible for the melanin biosynthesis pathway, and conidiation and methyl 5-(hydroxymethyl) furan-2-carboxylate toxin production, respectively. Molecular docking of compounds with THR and VelB proteins showed significant negative binding affinity using AutoDock4.2.3. Polyketide synthase plays an important role in the pathogenicity, which synthesised THR, another crucial step for the biosynthesis of DHN-melanin in both mycelia and conidia (Lu et al., 2022). The velvet family protein is reported to play a key role in the regulation of secondary metabolism, fungal growth, and sporulation in many filamentous fungi (Gao et al., 2017). The main member of the velvet protein family is VelB protein in a few fungal species, according to the literature (Bayram et al., 2008; Wiemann et al., 2010; Yang et al., 2013; Gao et al., 2017). Curcumin is observed to have the highest negative binding energy and hydrogen bonds with both THR and VelB proteins. Berberine and eugenol also showed good binding affinity with both virulence proteins, whereas α-pinene did not form hydrogen bonds with THR as well as VelB proteins and also the binding affinity was comparatively lower. The docking interaction indicated that the protein–ligand complexes that had good binding affinity were those that formed the highest number of hydrogen bonds (Shamsi et al., 2022). Curcumin-bound complexes of THR and VelB receptor proteins were employed to understand the stability and dynamical behaviour of the complexes. Also, 100 ns MD simulation analysis highlights the gain in the structural stability after binding of the curcumin molecules. In both complexes, the curcumin molecule remains stable in the corresponding binding site of the protein and forms one hydrogen bond with more than 80% of the time. This result suggests the possibility of designing a high-affinity inhibitor carrying the curcumin scaffold entity.

Plant-derived compounds (α-pinene, curcumin, berberine, and eugenol) are effective against *C. lunata*. The natural bioactive compounds possess less toxicity and associated side effects, which makes them a suitable candidate for drug discovery. Among all, curcumin exhibited effective antifungal properties to inhibit the growth of the fungus. Conidia adherence and their germination were restricted by the activity of these compounds. It was shown that the compounds down-regulate *brn1, velB*, *pks18*, and *clm1* gene expression, leading to decreased cell wall integrity and sporulation. The correlation between the virulence gene down-regulation and *in silico* molecular docking interaction represented the potential antifungal activity of bioactive compounds and highlighted virulence proteins THR and VelB as a possible drug target.

## Data Availability

The datasets presented in this study can be found in online repositories. The names of the repository/repositories and accession number(s) can be found in the article/Supplementary Material.
