# Supplementary material for: Gene expression, molecular docking, and molecular dynamics studies to identify potential antifungal compounds targeting virulence proteins/genes VelB and THR as possible drug targets against Curvularia lunata
Source: Front Mol Biosci. 2022 Dec 12;9:1055945. doi: 10.3389/fmolb.2022.1055945 (PMC9815619; doi:10.3389/fmolb.2022.1055945)
Supplement: Supplementary file 1 [file Presentation1.pdf]

Supplementary Document

Gene Expression, Molecular Docking and Molecular Dynamics Studies to Identify Potential Antifungal Compounds Targeting Virulence Proteins/Genes VelB and THR as Possible Drug Targets against *Curvularia lunata*

Himanshu Kamboj[1†], Lovely Gupta[1†], Pawan Kumar[2], Pooja Sen[1], Abhishek Sengupta[3*] and Pooja Vijayaraghavan [1*]

[1]Anti-mycotic Drug Susceptibility Laboratory, J3 block, Amity Institute of Biotechnology, Amity University, NOIDA, India
[2]School of Computational and Integrative Sciences, Jawaharlal Nehru University, New Delhi, India
[3]Systems Biology and Data Analytics Research Laboratory, Amity Institute of Biotechnology, Amity University Uttar Pradesh, Sector-125, NOIDA, Uttar Pradesh, 201301, India

[†]Equal Contribution and first authorship

[*]Corresponding authors

***Corresponding authors:**

Pooja Vijayaraghavan, PhD, Lab 120, J3 block, Amity Institute of Biotechnology, Amity University Uttar Pradesh, Sector-125, NOIDA, Uttar Pradesh, India

Tel: +919650548930; Email: vrpooja@amity.edu

Abhishek Sengupta, PhD, Lab 419B, J3 block, Amity Institute of Biotechnology, Amity University Uttar Pradesh, Sector-125, NOIDA, Uttar Pradesh, India

Tel: +918800662904; Email: asengupta@amity.edu

Authors' ORCID ID

Himanshu Kamboj- 0000-0002-2650-5563

Lovely Gupta- 0000-0002-2610-3142

Pawan Kumar- 0000-0002-8801-644X

Pooja Sen- 0000-0002-0819-1599

Abhishek Sengupta- 0000-0002-3958-3675

Pooja Vijayaraghavan- 0000-0001-5943-9462

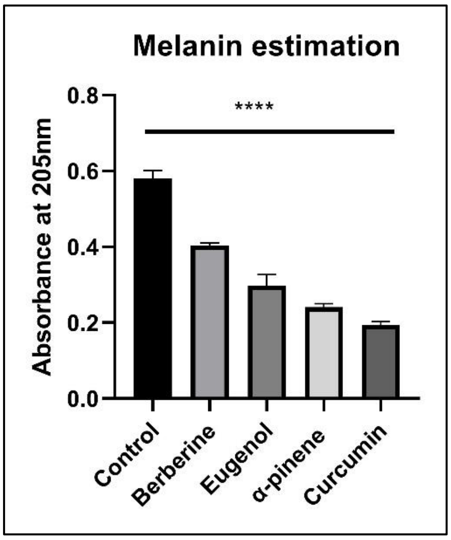

Figure S1. Effect of compounds (berberine, eugenol, α-pinene and curcumin) on *C.lunata* melanin using UV-visible spectrophotometer. Figure indicates reduction in melanin content after treatment with compounds. Curcumin treatment shows maximum reduction in melanin content followed by α-pinene, eugenol and berberine.

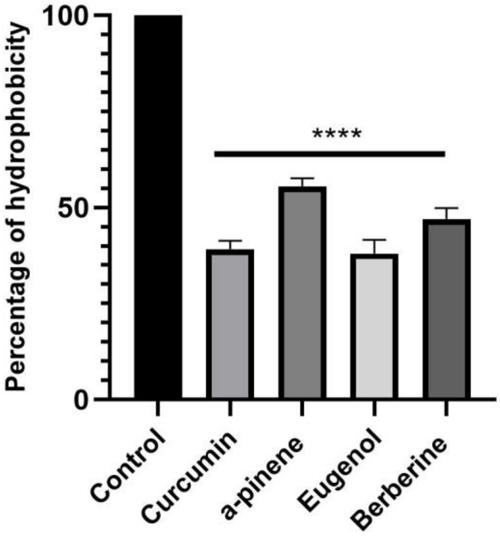

Figure S2. Effect of compounds (berberine, eugenol, α-pinene and curcumin) on cell surface hydrophobicity of *C.lunata* analysed at wavelength 630 nm. Figure represents significant reduction in CSH percentage between treated culture as compared to the control.

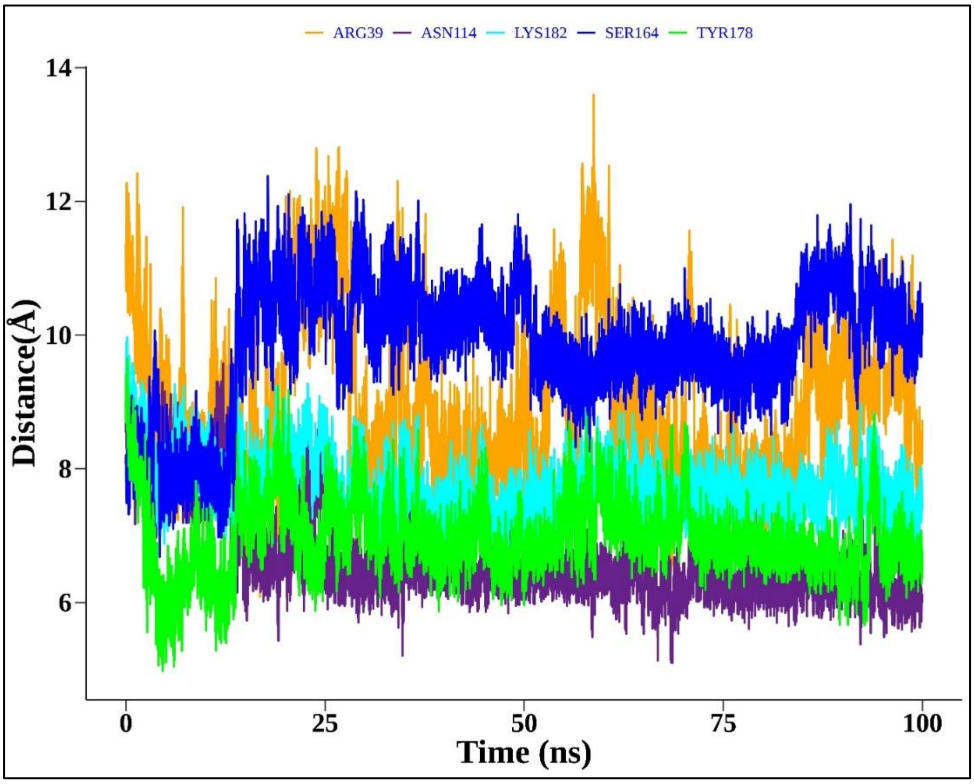

Figure S3: Distance between Curcumin and THR binding site residues (Arg39, Asn114, Lys182, Ser164, Tyr178). Despite the flexibility of the binding site residues, curcumin consistently maintains the distance after 25 ns.

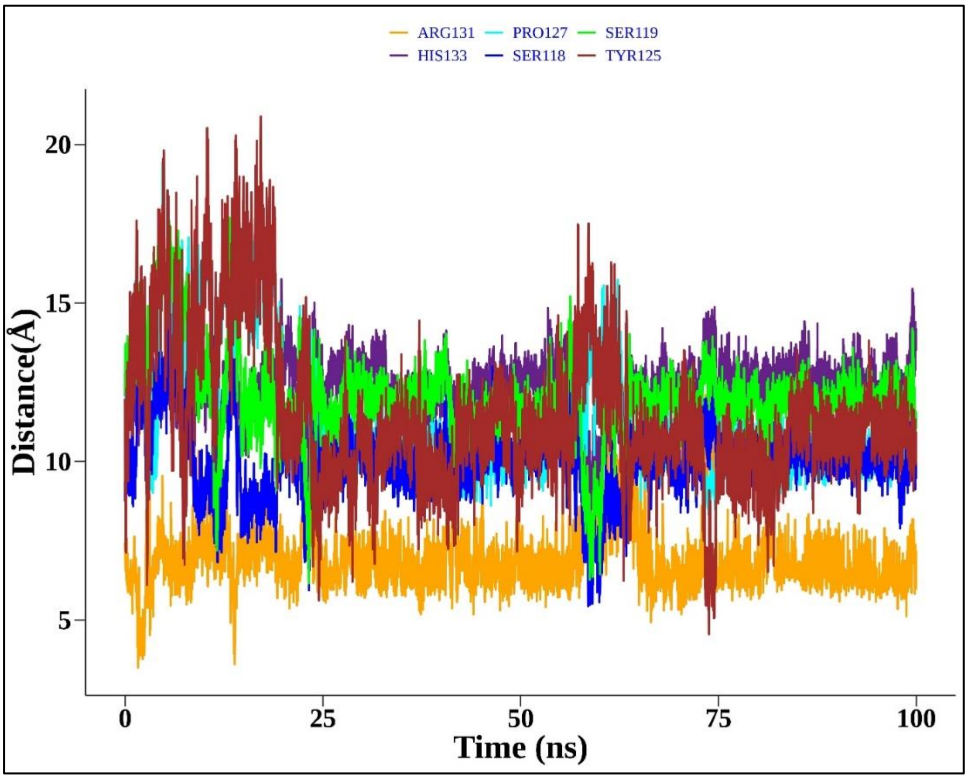

Figure S4: Distance between Curcumin and VelB binding site residues (Arg131, Pro127, Ser119, His133, Ser118, Tyr125). Despite the flexibility of the binding site residues, curcumin consistently maintains the distance after 25 ns.

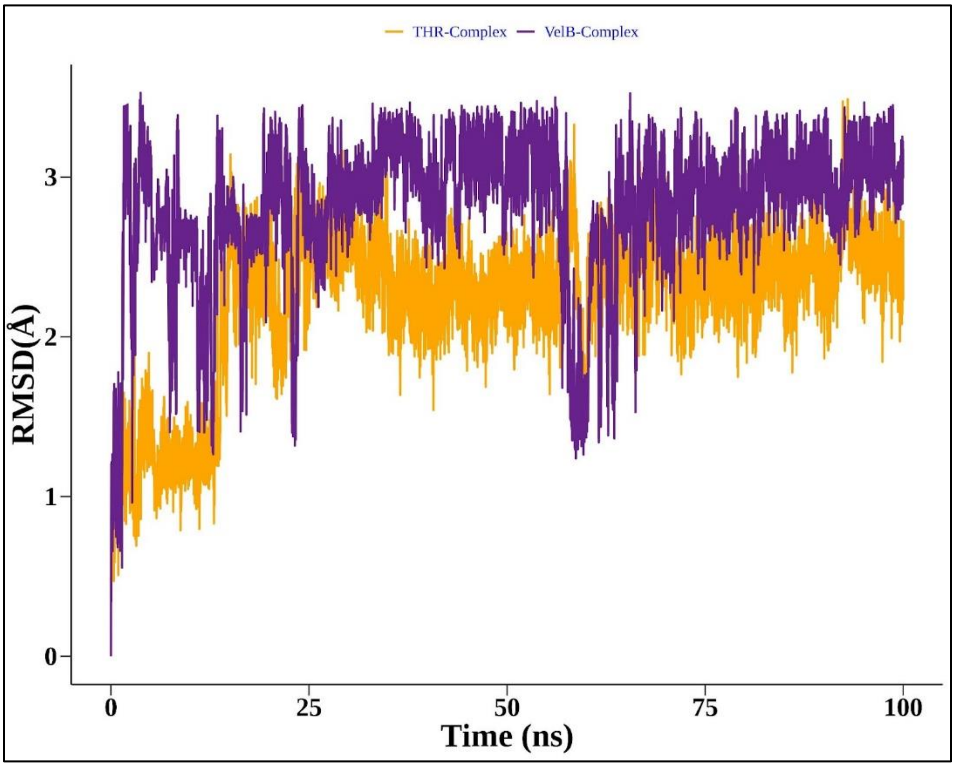

Figure S5: Curcumin RMSD in the binding site of THR (in orange color) and VelB (in purple color). In both cases, RMSD distribution of curcumin reflects that ligand molecule remain stable in the respective binding site of the receptor protein.
